# Scalable batch fabrication of ultrathin flexible neural probes using a bioresorbable silk layer

**DOI:** 10.1038/s41378-022-00353-7

**Published:** 2022-02-16

**Authors:** Clement Cointe, Adrian Laborde, Lionel G. Nowak, Dina N. Arvanitis, David Bourrier, Christian Bergaud, Ali Maziz

**Affiliations:** 1 LAAS-CNRS, 7 Avenue du Colonel Roche, F-31400 Toulouse, France; 2 CerCo, Université Toulouse 3, CNRS, Pavillon Baudot, CHU Purpan, BP 25202, 31052 Toulouse, France; 3 UMR Institut National de la Santé et de la Recherche Médicale 1048, Institut des Maladies Métaboliques et Cardiovasculaires, Toulouse, France

**Keywords:** Engineering, Environmental, health and safety issues

## Abstract

Flexible intracerebral probes for neural recording and electrical stimulation have been the focus of many research works to achieve better compliance with the surrounding tissue while minimizing rejection. Strategies have been explored to find the best way to insert flexible probes into the brain while maintaining their flexibility once positioned. Here, we present a novel and versatile scalable batch fabrication approach to deliver ultrathin and flexible probes consisting of a silk-parylene bilayer. The biodegradable silk layer, whose degradation time is programmable, provides a temporary and programmable stiffener to allow the insertion of ultrathin parylene-based flexible devices. Our innovative and robust batch fabrication technology allows complete freedom over probe design in terms of materials, size, shape, and thickness. We demonstrate successful ex vivo insertion of the probe with acute high-fidelity recordings of epileptic seizures in field potentials as well as single-unit action potentials in mouse brain slices. Our novel technological solution for implanting ultraflexible devices in the brain while minimizing rejection risks shows high potential for use in both brain research and clinical therapies.

## Introduction

Chronically implanted microelectrodes have been a key tool in neuroscience research by allowing the recording of electrical brain activity at the level of a small population of neurons (local field potential (LFP), multiunit spiking activity) and of individual neurons (single-unit activity). The past decades have seen impressive technological developments of neural implants incorporating electrodes at the micrometer scale, e.g., silicon-based pin (Utah), flat (Michigan), or wire (floating microwire arrays), for the characterization of neuronal activity^1–3^. Such devices are now routinely used in animal studies^4^. Although long-lasting recording is sometimes achieved using these probes^5^, large variations in electrical recording capabilities have often been reported^6^. The implementation of long-lasting intracerebral recordings is limited by the lack of stability at the interface between conventional electrodes and brain tissue^7,8^. This is partly due to the mechanical mismatch between the stiffness of the materials, e.g., silicon, glass, platinum, or iridium (Young’s modulus *E* ≈ 150 GPa), constituting such probes and the softness of the cerebral tissue (*E* ≈ 10 kPa)^9^. This mechanical mismatch, which can be as large as seven orders of magnitude, leads to irreversible tissue damage and glial scar formation, resulting in failure of the device within months or even weeks after implantation^10,11^.

To improve the brain tissue-electrode interface, research has focused on the use of flexible probes, which would achieve better compliance with the surrounding neural tissue and minimal rejection^10,12^. The fabrication of these compliant devices typically involves either the use of soft polymeric materials as substrates, e.g., parylene^13–15^, polyimide^16,17^, polydimethylsiloxane (PDMS)^18^, hydrogels^19^, and/or the use of significantly thinner stiff materials^20,21^. However, an important issue with flexible probes is that they have a tendency to fail penetrating the brain meninges and reach their location goal. Indeed, a device that is too soft tends to bend when pressed against a rigid surface, such as the pia mater^12^. Strategies have been explored to find the best way to implant flexible probes into the brain while still maintaining their flexibility once positioned. Some teams have focused on the use of a stiff shuttle^20,22,23^ that is removed immediately after implantation, while others have promoted the integration of a stiff bioresorbable coating that is not removed but dissolves inside the brain on a time scale of minutes to days^24,25^. Due to the tissue trauma caused by the implantation and withdrawal of a stiff shuttle, the integration of bioresorbable coatings as a temporary stiffener has been shown to better address both mechanical and biological failures^26^. Various bioresorbable polymers, e.g., poly(ethylene glycol) (PEG), polylactic acid (PLA), chitosan, and silk fibroin, have been reported to be excellent candidates to add to polymeric implants for facilitating insertion into the brain^12^. In addition, they benefit to some extent from common attributes, such as a high Young’s modulus, proven biocompatibility for in vivo application purposes, and resorption when in contact with biological tissues^27^.

Although the reported flexible implants incorporating a biodegradable coating have shown successful short-term electrical recordings, their fabrication process is incompatible with standard microfabrication techniques. Manual handling is required, which limits further downscaling (to less than 10 µm) and makes it difficult and time-consuming to create many devices in parallel^12,28,29^. Furthermore, existing flexible probes require the additional preparation of a carrier support for the biodegradable coating, with a manual assembly procedure that increases the difficulty of reducing the dimensions and volume of the probe. These coated probes may generate surgical footprints of a volume considerably larger than the electrode itself, which may induce trauma to brain tissue in the range of hundreds of micrometers to millimeters^24,25^. Cellular and/or vascular damage may then elicit sustained inflammation and tissue responses. A global rethinking of the production framework is therefore needed to effectively achieve the simple integration of biodegradable coatings to allow further development of minimally invasive neural probes.

To achieve this goal, we report here a versatile fabrication framework utilizing a bioresorbable silk fibroin layer that can be integrated in a microfabrication process for preparing ultrathin parylene-based penetrating probes. The probes consist of a silk-parylene bilayer, which is obtained by successively depositing layers on top of each other. The first layer is obtained using a degradable silk fibroin coating as a temporary stiffener that allows for the insertion of ultrathin parylene-based flexible devices deep into the brain. The additional subtlety of the process derives from the exposure of the silk fibroin layer to methanol, which increases the crystallized domains in the film, allowing the degradation of the stiffening layer through proteolytic reactions within a programmable time lapse. Furthermore, insertion trials in artificial brain phantoms are achieved without buckling of the probe or undesired alteration of the electrical properties of the electrodes. We also demonstrate successful ex vivo insertion of the probe with high-fidelity acute recordings of epileptic activity as well as single-unit action potentials in mouse brain slices. The possibilities offered by our approach are very promising for the development of ultraflexible probes for application in research and brain therapy.

## Results and discussion

### Development of the bilayered probes

**Fig. 1:**
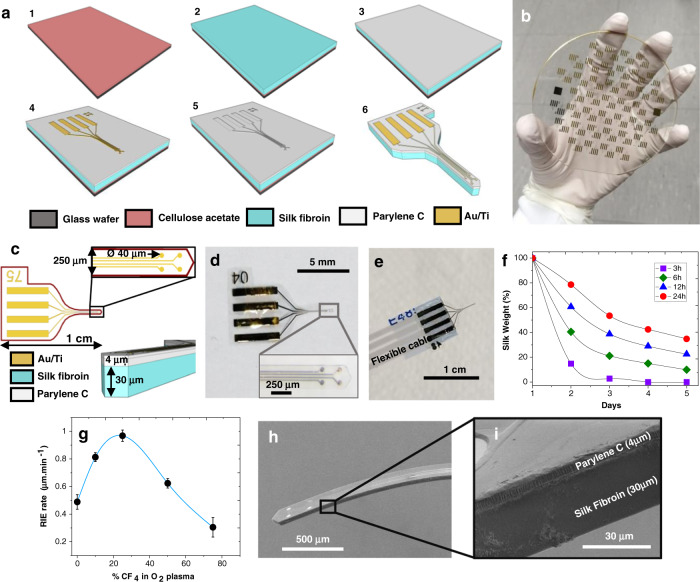
Fabrication of the bilayered silk-parylene probes. **a** Schematic illustration of the main fabrication steps on a glass substrate: (1) cellulose acetate deposition, (2) silk fibroin deposition, (3) parylene base layer deposition, (4) gold microelectrode patterning, (5) parylene top layer deposition and (6) final shaping of the silk-parylene probe by RIE and release from the substrate. **b** Picture of the 4-inch glass substrate after batch microfabrication. The substrate contains 80 elements (less than 1 cm each). **c** Schematic illustration of the bilayered silk-parylene probe with characteristic dimensions. The device contains four recording microelectrodes with diameters of 40 μm patterned on a 3 mm-long and 250 μm-wide shank. **d** Picture of the microfabricated implant. The inset shows the corresponding magnified view of the 4 gold microelectrodes. **e** Picture of the silk-parylene probe highlighting the contact pads bonded with the FFC cable for the following electrochemical and electrophysiological measurements. **f** Enzymatic degradation of silk fibroin after different immersion times in methanol. **g** Etching rate of silk fibroin vs. percent of CF_4_ in O_2_ plasma at 500 W and 20 mTorr. An optimal ratio of 25/75 was found. **h** SEM image of the final bilayered silk-parylene shank, and **i** the corresponding magnified SEM image

Our ultrathin flexible probes are developed using standard microsystem techniques. The probes are made of a silk-parylene bilayer that comprises four gold microelectrodes coated with the conducting polymer poly(3,4-ethylenedioxythio-phene):poly(styrene-sulfonate) (PEDOT:PSS) to lower the impedance and obtain a better signal-to-noise ratio for neuron recording^13,30^. The schematics of the fabrication procedures and the probe structure are shown in Fig. 1a. Briefly, there are three main fabrication stages. In stage I, a cellulose acetate (CA)-coated glass substrate is coated with a silk fibroin layer before (stage II) an ultrathin parylene-based structure is microfabricated on top of the silk layer using standard top-down lithography techniques (discussed in more detail later in the paper). Finally, in stage III, the whole silk-parylene probe undergoes microscale shaping by a reactive ion etching (RIE) process before being released from the glass substrate.

There are a couple of key considerations in this design. First, the silk fibroin material is chosen among other bioresorbable materials on the basis of its excellent biocompatibility, tunable biodegradability, and high mechanical strength^31^. Indeed, silk fibroin possesses a Young’s modulus of *E* ≈ 3 GPa so that the thickness of parylene-C can be drastically reduced to the µm level while allowing for the handling and implantation of the probe. The biodegradable silk layer is described in detail below because of its specific chemical structure and its importance in the final device. Active research is currently underway on the use of silk biomaterials, particularly to obtain a biodegradable coating for implantable neural recording devices^12^. However, no work has yet been reported on the integration of degradable biopolymers in the batch fabrication of neural recording devices, allowing for further scaling down (less than 10 μm thick) and preventing *de facto* any manual handling procedure and buckling issues during implantation.

The thickness of the silk layer can be tuned by adjusting the polymer dilution percentage or the volume of the solution on the casting area. As an example, a dose of 0.1 mL/cm^2^ of 7 wt.% silk fibroin solution produces films with a thickness of ∼30 ± 5 μm (± SD).

Silk degradation time is another critical parameter. Depending on the implantation strategy, the probe position may need to be adjusted for a prescribed time of several minutes or more, during which it must remain stiff. This critical time is determined by the degradation rate of the bioresorbable coating. The lifetime of the bioresorbable layer can be specifically adjusted via the crystallinity of the silk protein, i.e., the β-sheet content^32^. Indeed, treating silk by immersion in methanol increases the β-sheet content in the film. In a proteolytic medium (protease XIV (PXIV) in PBS solution (1 U/ml)), silk fibroin samples degrade at different rates depending on the methanol treatment time. For instance, we find that films treated for 3 and 6 h degrade within a few hours while those treated for 12 and 24 h last up to one week (Fig. 1f). This control over the degradation time is consistent with the literature, where reports have shown proteolytic degradation in vitro of water-stable silk films after approximately two weeks^25,32^.

The second step consists of fabricating an ultraflexible parylene-based probe on top of the silk fibroin substrate (Fig. 1a) (more details are available in the Materials and Methods). Parylene is chosen as the substrate for its well-documented biocompatibility, chemical inertness, and high electrical and moisture insulation properties. Figure 1c shows the design of the ultraflexible parylene-based probe, which consists of three layers: a top parylene layer (thickness: 1 μm) for encapsulation, a middle Au layer (thickness: 200 nm) for the electrophysiological measurement, and a bottom parylene layer (thickness: 3 μm) for mechanical support. The total thickness of the probe is only 4 μm.

The shaping of the silk-parylene probe is achieved by dry etching, which is performed through RIE using a 75/25 O_2_/CF_4_ gas ratio. Under these conditions, the microfabrication of bilayered silk-parylene microstructures can be performed with precise control of the geometry, size, and shape, as illustrated in Fig. 1d, h and i. The proof of concept presented in Fig. 1b contains 80 elements, and each device contains four recording gold microelectrodes with diameters of 40 μm patterned on 3 mm-long and 250 μm-wide probes. In other words, large-scale batch fabrication of precisely defined silk-parylene probes can be performed directly on the glass substrate. Finally, the cellulose acetate sacrificial layer is dissolved in acetone to release the bilayered silk-parylene probes from the substrate (Fig. 1d). The probe is robust enough to easily bond directly with external electrical connections (Fig. 1e).

### Insertion testing in brain phantom

**Fig. 2:**
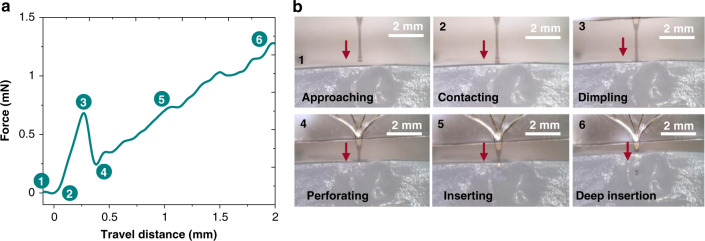
Insertion test of the silk-parylene probe in brain phantoms. **a** Force profile during insertion of the shank in 1 wt.% agarose gel (Young’s modulus of 40 kPa), and the numbers correspond with those in (**b**). **b** Optical pictures showing the different stages of insertion in correlation with the evolution of the force

Our bilayered probes are designed as insertable ultrathin flexible devices that do not buckle during insertion in the brain. An important parameter lies in the mechanical stability of the stiffening resorbable coating during the implantation/explantation process. To test the rigidity afforded by the coating, we performed cyclic insertions of the probe into a brain tissue-mimicking phantom (1 wt.% agarose gel, Young’s modulus of 40 kPa). Figure 2b shows the whole process of implanting the probe into the brain tissue-mimicking gel, showing (1) the probe mounted on the manipulator approaching and (2) contacting the gel, (3) the probe successfully perforating the gel, (4) the probe inserted in the gel, and (5 and 6) the probe deeply inserted into the gel. The bilayered silk-parylene probes thus succeed in penetrating the brain phantom without any buckling or bending, which clearly indicates that the insertion shuttle is sufficiently stiff. From the insertion experiment (Fig. 2a), the first peak force (stage 3) is 0.7 mN, corresponding to the minimum force required to penetrate the tissue-mimicking gel. Other works show a similar minimum force^25,33^. As Movie S1 (Supporting Information) shows, the probe is explanted from the gel without mechanical damage and successfully reinserted into the gel a second time. It is worth mentioning that the bare parylene shank itself, being only 4 µm thick, curls and cannot be handled or manipulated without the bioresorbable silk polymer support.

The type of brain phantom we used has been widely used as a model of implantation in gray and white matter^25,34^, yet it does not take into consideration other brain features. Indeed, the minimum insertion force also depends on animal species, on the biological tissue (pia or dura mater, gray or white matter), on the size and shape of the shank, on the speed of approach, etc^34^. For example, higher insertion forces are needed for dura mater penetration^34^. Our fabrication technology allows complete freedom in terms of materials, size, shape, and thickness. Thus, the probe buckling strength can be easily tuned depending on the biological target.

We next performed in vivo implantation in an anesthetized mouse to evaluate the ability of the silk-stiffened probe to achieve more difficult tasks of penetration, e.g., through the pia mater. The dura mater is resected prior to implantation. The probe is lowered with a manual micromanipulator through the pia mater and is left in the somatosensory cortex for one hour after insertion, such that the silk coating has enough time to dissolve. Figure S4 shows images of the silk-coated probe during insertion in the mouse brain. Insertion of the probe occurs with no sign of buckling, which is consistent with what we observed in the in vitro insertion tests on the gel brain phantoms (Fig. 2). We do not notice any visible damage to the electrodes after insertion and retraction. These results indicate that our probe design is compatible with in vivo implantation.

### Standard compression tests

**Fig. 3:**
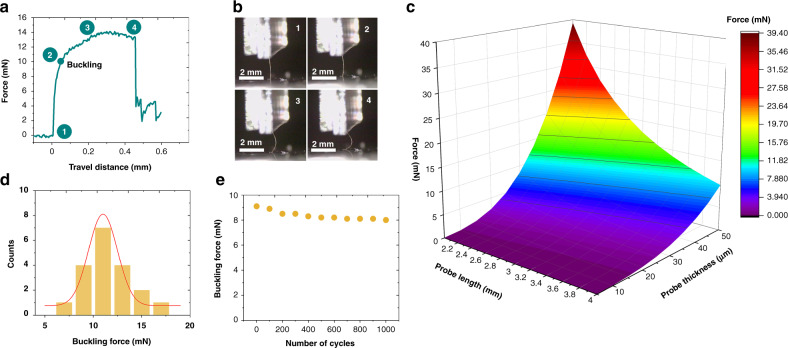
Compression tests of the bilayered silk-parylene probes against a hard substrate. **a** Force profile during compression, numbers and buckling position, which correspond with those in (**b**). **b** Series of optical pictures showing the corresponding stages of shank compression in correlation with the evolution of the force. **c** Theoretical influence of the shank length (in mm) and thickness (in µm) on the buckling force. The model was clamped-pinned, the cross-section of the device was considered a constant rectangle, and the shanks were beams fixed on one side and pinned on the other side (*K* = 0.7). We assumed that *E* for parylene C and silk fibroin was 3 GPa. **d** Electrode-buckling force histogram of the silk-parylene probes (*N* = 20). **e** Monitoring of the average buckling force as a function of the number of bending cycles (1000 cycles) with a bending radius of 0.3 mm

We further studied the mechanical properties of our bilayered probes against a hard substrate. Axial compression of beams eventually results in their buckling. The highest force that a sample can withstand before bending is called the buckling force *F*_buckling_. As depicted in Fig. 3a, compression tests on a hard substrate show the clear superiority of the bilayered silk-parylene assembly in terms of its mechanical strength. The silk coating improves the probe strength, with an average buckling strength of 10.9 ± 1.3 mN (Fig. 3d), which is 15 times higher than the force required to penetrate the brain-mimicking gel (Fig. 2a). These experimental values were compared with theoretical analysis predictions (Fig. 3c). Our probes are modeled as single beams, whose buckling force is defined by Euler’s formula: *F*_buckling_ = *π*^*2*^*I*_*x*_*E/(KL)*^2^. The buckling force along the *x*-axis of a clamped beam is linked to its Young’s modulus *E*; the area moment of inertia *I*_*x*_ (*I*_*x*_ = *wt*^3^*/12*) along the *x*-axis; the length *L*, width *w*, and thickness *t* of the probe; and the effective length factor of the column *K*. The cross-section of the device is considered a constant rectangle, and the shanks are beams fixed at one side (*K* = 0.7). Assuming that the *E* values for parylene and silk fibroin are 3 GPa^29,33^, the calculation yields a theoretical buckling force of 7.2 ± 2.3 mN for the probe (Fig. 3c). The calculated theoretical buckling force predicts the trend and order of magnitude of the experimental values (Fig. 3d).

In addition to axial compression tests, the mechanical stability of our probes under long-term cyclic compression was tested under cyclic loading with a bending radius of 0.3 mm. As shown in Fig. 3e, the original buckling force (first cycle) is 9.1 ± 0.5 mN and shows minimal change (only a 10% decrease) after nearly 1000 bending cycles. After prolonged axial compression tests, the bilayered silk-parylene probes do not show any peeling or sign of delamination. This indicates that these engineered bilayered silk-parylene probes can endure a very large number of contact cycles without any damage or delamination.

### Electrical and electrochemical characterization

**Fig. 4:**
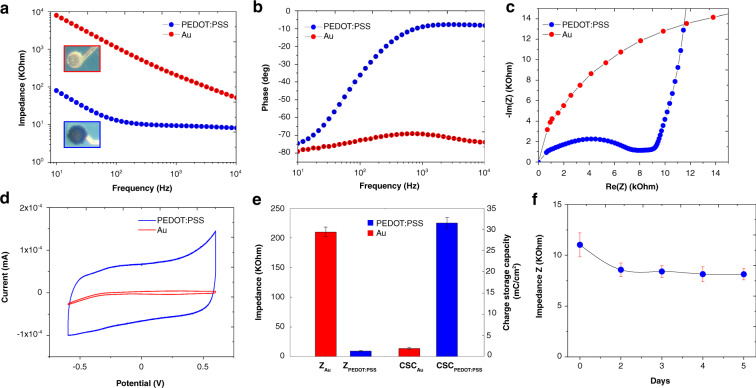
Electrochemical characterization of the microelectrodes. **a** EIS measurements of the gold- and PEDOT:PSS-coated microelectrodes in PBS at room temperature from 10 Hz to 7 MHz. **b** Corresponding phase vs. frequency plotting of gold- and PEDOT:PSS-coated microelectrodes. **c** Nyquist diagram at frequencies ranging from 10 Hz to 7 MHz of gold- and PEDOT:PSS-coated microelectrodes. **d** CV in the PBS buffer at room temperature by potential sweeping between −0.6 and +0.6 V at 200 mV/s vs. the Ag/AgCl reference electrode of gold- and PEDOT:PSS-coated microelectrodes. **e** Comparison of the electrochemical characteristics (Z at 1 kHz and CSC) between the gold- and PEDOT:PSS-coated microelectrodes. **f** Impedance evolution at 1 kHz of the PEDOT:PSS-coated microelectrode in PBS-soaked gel, obtained from the EIS measurements

Possible alteration of the electrical and/or electrochemical properties of the probe should also be investigated to ensure their inherent electrode performance. We evaluated the electrical properties of the microelectrodes by electrochemical impedance spectroscopy (EIS), cyclic voltammetry (CV), and calculations of the charge storage capacity (CSC). Figure 4a, b shows the Bode plots across the frequencies of interest (10 Hz–7 MHz) for a gold microelectrode with a diameter of 40 µm before and after the electrochemical deposition of PEDOT:PSS. The mean impedances at 1 kHz are used for comparison, as action potentials have a characteristic frequency band centered at that frequency (Fig. 4e). Before PEDOT:PSS deposition, the average impedance is 210 ± 8.2 kΩ (*n* = 5) in PBS, while the mean impedance decreases to 9.4 ± 0.9 kΩ after polymer deposition. This well-known phenomenon is due to an increase in the effective surface area with the formation of PEDOT:PSS material, leading to a decrease in impedance of the microelectrode. The corresponding phase plot of the impedance reveals that the PEDOT:PSS microelectrode is capacitive in the low frequency range (10 Hz) and more resistive at higher frequencies (Fig. 4b). The Nyquist plot recorded in PBS is presented in Fig. 4c. The deposition of PEDOT:PSS produces a very small radius of the semicircle on the Nyquist plot with a charge transfer resistance of approximately 7.8 kΩ, revealing the low electron-transfer resistance associated with the polymer coating.

We next evaluated the charge transfer capabilities of the microelectrodes. CV from −0.6 and 0.6 V at a scan rate of 0.2 V/s was performed, and the cathodal CSC was calculated by the time integral of the cathodal currents within the cycled region (Fig. 4d). The cathodic CSC increases from an average value of 1.95 ± 0.3 mC cm^−2^ for the gold microelectrodes to 31.5 ± 1.3 mC cm^−2^ for the PEDOT-coated microelectrodes. A higher charge capacity results in higher charge injection, which is desirable for electrical stimulation.

In addition, to track the integrity of the probe structure and electrical connections, in vitro EIS measurements were carried out daily for a 5-day period in saline brain phantoms (gels soaked in PBS) with four different probes. Figure 4f shows the impedance measured at 1 kHz after the insertion of the silk-parylene probe in PBS-based gel as a function of immersion time. EIS measurements over the 5-day period demonstrate a small decrease (~11 ± 1.2–8 ± 0.5 kΩ) followed by a stabilization of the impedances. We observe no distortion of the probe upon repeated insertion into the gel, which indicates that the thickness of the silk layer remains sufficient to carry the full ultrathin structure without compromising the electrical integrity of the parylene probe. The stable electrical impedance throughout the process proves the robustness of our design and protocol for in vivo implantation.

### In vitro proteolytic degradation

**Fig. 5:**
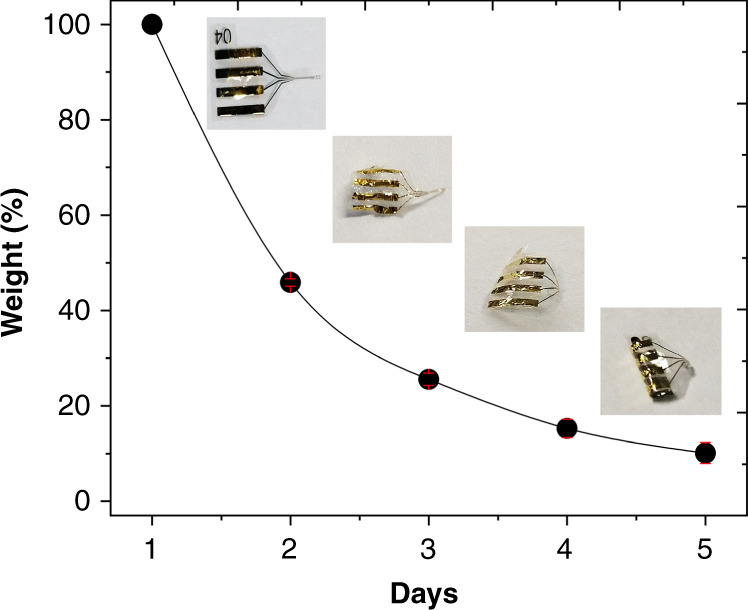
In vitro proteolytic degradation of the silk layer. Enzymatic degradation of the silk in the bilayered silk-parylene probes by protease XIV (PXIV) solution (1 U/ml) for 5 days at 37 °C

To track the integrity of the structure, we performed incubation studies in PBS containing the enzyme protease XIV (Fig. 5). For this experiment, probes are crystallized for 6 h in methanol. Most naturally biodegradable polymers degrade in vivo via enzymatic degradation, owing to the stability of their backbone structure. It is generally accepted that silk protein is degraded mainly by protease^35^. Protease XIV has been widely used to mimic extracellular degradation mediated by proteolytic enzymes. However, in vivo, the degradation of silk depends on both implant-related factors (molecular weight, molecular structure, crystallinity, etc.) as well as on host-related factors (i.e., immune response at the implant site)^35^. The silk biodegradability of the silk-parylene probes is measured as the loss of weight of the implant during continuous incubation at 37 °C in proteolytic solution for one week. In the proteolytic environment, the silk-parylene probes demonstrate a gradual decrease in mass, corresponding to slow protein fragmentation during incubation. This experiment shows the importance of using a biodegradable silk coating as a temporary stiffener to deliver ultrathin parylene-based flexible devices in deep tissue. It is worth mentioning that the bare parylene shank, which is only 4 µm thick, curls and cannot be handled or manipulated without the mechanical silk support.

### Electrophysiological recordings from mouse brain slices

**Fig. 6:**
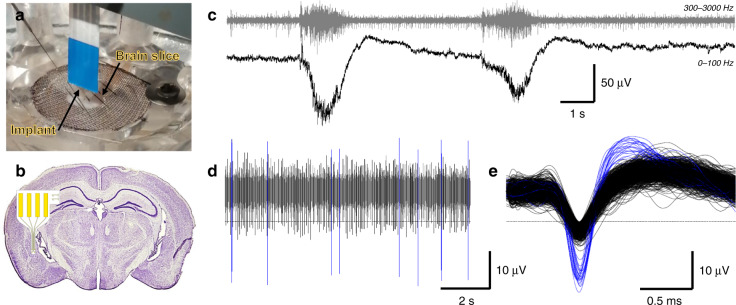
Electrophysiological recording in the piriform cortex of a mouse brain slice. **a** Photograph of a 35 μm-thick silk parylene probe inserted into a mouse brain slice: insertion occurs with no sign of buckling. **b** Scheme of the placement of the silk-parylene neural probe. **c** Traces show two consecutive epileptic seizures. The upper trace corresponds to the bandpass filtered signal (300–3000 Hz) used to reveal spiking activity, and the bottom trace corresponds to the low pass filtered signal (cutoff at 100 Hz) used to reveal slow changes in the LFP. **d** Traces show spontaneous spiking activity occurring between epileptic seizures. The dashed line indicates the threshold used for sorting action potentials. It is set at −4xRMS of the voltage trace (RMS calculated outside of the epileptic seizure). The action potentials that cross the threshold are examined through cluster analysis and interspike interval analysis. **e** Overlap of the action potentials recorded over 5 min at high temporal resolution. The constancy of the spike shape and refractory period (6.4 ms in that case) definitively ensures that the blue action potentials belong to one single unit. The action potentials in black correspond to multiunit activity

Electrophysiological experiments were conducted on mouse brain slices to assess the recording quality of the probes. The recordings were performed after the silk coating had completely dissolved to ensure that the 4 µm-thick flexible parylene probes were still functional for acute recordings. The silk coating process was tuned so that the coating would completely dissolve in the slice within 30 min (corresponding to a 1 h long immersion of the probe in methanol for crystallization). Probe insertion was aimed at layer 2 of the piriform cortex (Fig. 6b). This layer is characterized by a high density of neuronal cell bodies. As spontaneous activity is low or null in most regions of the mouse brain ex vivo, we added 4-aminopyridine (4AP, 100 µM) to the superfusion solution to activate the slices. 4AP infusion usually leads to the appearance of spiking activity and most often induces epileptiform activity^36^. The latter provides an opportunity to report on the capability of the probe to record LFPs in addition to single- and multiunit spiking activity. Examples of epileptic and spiking activities are presented in Fig. 6c–e. Epileptic activity is characterized by the occurrence of epileptic seizures in the LFP, which are evidenced by low pass filtering (0–100 Hz) of the voltage trace (Fig. 6c, bottom trace). Bandpass filtering (300–3000 Hz) of the same original trace reveals the action potentials of neurons (Fig. 6c, top trace). Large bursts of action potentials can be observed at the time of epileptic seizures. Action potentials can also be seen between epileptic seizures (Fig. 6c, d). We examined whether single-unit activity, i.e., action potentials that can be attributed to the activity of one single neuron, could be observed in these periods. For this purpose, portions of traces (2 ms wide, Fig. 6e) were extracted using a threshold set at −4xRMS of the voltage trace (dashed lines in Fig. 6d, e). These were then submitted to PCA and clustering (not illustrated). This analysis allows for segregation of different spike shapes. The spike shapes belonging to a given cluster are attributed to one single unit if the interspike interval distribution histogram (not illustrated) shows a clear refractory period, i.e., no interval <1 ms. Otherwise, the cluster corresponds to multiunit activity, that is, action potentials that are issued from at least two neurons and that cannot be segregated. The blue action potentials in Fig. 6e correspond to the activity of one single unit, identified as such by its constant spike shape and a refractory period of 6.4 ms. The action potentials in black correspond to multiunit activity. Overall, multiunit activity is recorded in all trials, and single units are recorded in 7/12 trials.

## Conclusion

This work proposes a novel and versatile approach to fabricate, pattern, and deliver ultrathin probes consisting of a silk-parylene bilayer. The biodegradable silk layer provides a temporary stiffener that can be used to deliver ultrathin parylene-based flexible devices in deep tissue. Our innovative and robust batch fabrication technology allows complete design freedom of the probe in terms of materials, size, shape, and thickness. We systematically studied the behavior of the bilayered structure in gel brain phantoms and demonstrated that parylene probes as thin as 4 μm could be delivered accurately to a desired depth with intact geometry and electrical functionality. We also demonstrated successful insertion of the probe in the mouse brain in vivo. Insertion in ex vivo mouse brain slices allowed acute recordings of LFP, as well as spontaneous multi and single-unit activities. These results suggest that our design provides a versatile technological solution for producing ultraflexible and ultrathin devices that might be implanted in the brain with minimal rejection. In future works, we will directly address this issue by performing in vivo neural recording and by correlating the quality of the recording with the presence or lack of inflammatory reaction and glial scar formation.

## Materials and methods

### Chemicals

Parylene C dimer (PXC) was purchased from Comelec SA. ECI 3012 photoresist was purchased from Microchemicals GmbH. MF CD26 developer was purchased from MicroChem. 3,4-Ethylene dioxythiophene (EDOT), poly(sodium 4-styrenesulfonate) (NaPSS), and acetone were purchased from Sigma Aldrich. Phosphate-buffered saline (PBS, Gibco DPBS 1X) was purchased from Fisher Scientific. Platinum (Pt) counter electrodes and silver/silver chloride (Ag/AgCl) reference electrodes were purchased from WPI. Solutions were prepared with deionized water (18 MΩ).

### Bioresorbable silk fibroin solution preparation

Silk fibroin aqueous solution was prepared from Bombyx mori cocoons following a protocol detailed previously^31^. The fibroin protein was first extracted from the silk fibers by boiling the *silk* cocoons (5 g) in a solution of 0.02 M Na_2_CO_3_ for 30 min. The regenerated silk fibroin was then recovered and rinsed thoroughly in deionized water before being dried overnight under ambient conditions. The dried silk fibroin (3.6 g) was dissolved in a 9.3 M LiBr solution at 60 °C for 4 h. The salt was then removed by dialyzing the solution against deionized water for 24 h at room temperature using a dialysis membrane (MWCO 3.5 KD, Spectra/PorTM) and regularly changing the water. Centrifugation was performed to remove impurities. The recovered silk fibroin solution had a final concentration of 7 wt.%.

### Probe microfabrication

A 4-inch glass wafer was used to prepare the overall process. The glass substrate was first cleaned by MW-oxygen plasma (800 W, 10 min, O_2_) prior to processing. The fabrication began with the deposition of a cellulose acetate layer (~2 μm) by spin-coating at 1000 rpm for 30 s (5% w/vol in acetone). It acted as a sacrificial layer to release the final device from the substrate. The silk fibroin aqueous solution (7 wt.%) was deposited by drop casting and left to dry under ambient conditions overnight, resulting in an ~30 µm-thick silk film. The thickness of the resulting film was controlled by adjusting the volume of the silk fibroin solution. Then, a 3 μm-thick layer of Parylene C (PXC) was deposited onto the silk-coated substrate through CVD using C30S Comelec equipment. A 50/200 nm layer of Ti/Au was then deposited by evaporation and patterned through an electroplated nickel-based shadow mask. Another 1.3 μm top layer of Parylene C was deposited onto the processed metal layer. The shape of the electrode pads, contacts and device body were defined by photolithography steps followed by RIE in O_2_/CF_4_ plasma (75/25) at 500 W and 20 mT. Finally, the cellulose acetate sacrificial layer was dissolved with acetone to release the bilayered silk-parylene probes.

### Electrochemical characterization

Electrochemical characterization was performed with a 3-electrode system that included a Pt wire as the counter electrode, a Ag/AgCl wire as the reference electrode and the gold microelectrodes from the silk-parylene probe as the working electrodes. EIS and cyclic voltammetry (CV) were performed using a Bio-Logic VMP3 potentiostat. CV was performed in PBS at room temperature by potential sweeping between −0.6 and +0.6 V at 200 mV/s vs. Ag/AgCl reference, allowing cathodal charge storage capacity (CSCc) evaluation^37,38^. EIS was also performed in PBS at room temperature by applying a 10 mV sine wave at frequencies ranging from 10 Hz to 7 MHz. Improved electrical properties were achieved by PEDOT:PSS deposition. CV was performed in EDOT:NaPSS solution (10 mM:34 mM) at room temperature by potential sweeping between −0.7 and +1 V at 10 mV/s vs. Ag/AgCl reference. CV and EIS were then performed again to compare the evolution of the CSCc, impedance, phase, and Nyquist results.

### Crystallization procedure of the silk fibroin

Water-insoluble silk films were prepared by methanol treatment, which increased the crystallized domains in the film, allowing degradation within a programmable time window. The bilayered silk-parylene probes were positioned between two flexible filter papers and held in position with clips to prevent any deformation of the devices during annealing. The probes were then immersed in 80% methanol solution at room temperature for a fixed time to increase the β-sheet crystal content. After methanol annealing, the filter paper was removed, and the implants were dried under vacuum at 40 °C for 1 h to remove all traces of methanol.

### Standard compression tests

Standard compression tests against a hard substrate were performed to assess the mechanical properties of the bilayered silk-parylene probes. The axial compression of the implant allowed us to evaluate its buckling force, which corresponds to the highest force that a sample can withstand before bending. To guarantee the stability of the test, the implants were held between two glass plates, and compression was carried out over a tip length of approximately 3.4 mm. The silk-parylene probes were fixed on a MARK-10 ESM303 test bench coupled with a MARK-10 M5-012 force sensor. An internally developed LabVIEW program allowed the equipment to be controlled and the force to be monitored according to the displacement of the implant. Compression tests were performed at a slow speed of 2 mm min^−1^ for optimal monitoring of the buckling force. A video recording (Movie S1) of the compression test using a Dino-Lite Edge camera was made to complete the experiment.

### In vitro insertion into the gel brain phantoms

Insertion tests of the probes were performed using 1% w/v agarose gel brain phantoms imitating the mechanical properties of brain tissue. In the same way as in the compression tests, the silk-parylene probes were held between two glass plates and fixed to the MARK-10 test bench. To limit damage during the insertion tests and to have optimal monitoring of the forces involved, the experiment was carried out at a very low speed of 0.5 mm min^−1^. A video recording was made using the Dino-Lite Edge camera to observe the different stages of insertion in correlation with the evolution of the force.

### Dissolution tests

To assess the biodegradation of the silk fibroin layer, an enzymatic degradation test was carried out over several days. Briefly, five implants (~2 mg) were selected and incubated in 1 ml of protease (Proteas XIV from *S. griseus*, 3.5 U/mg, Sigma-Aldrich) and PBS (1 U/ml of PBS buffer) at 37 °C. The implants were photographed and weighed every day after cleaning with DI water and dried at 60 °C for 10 min. The enzyme solution was changed after each weighing to maintain enzymatic activity.

### Brain slice preparation

All procedures were conducted in accordance with the guidelines from the European Community (directive 2010/63/UE) and from the French Ministry of Agriculture, Agri-food and Forestry (décret 2013–118) and were approved by the Ministère de l’Enseignement Supérieur, de la Recherche et de l’Innovation (N° 15226-2018052417151228). Two- to 4-month-old C57BL/6 wild-type female mice were used for brain slice preparation. The protocol has been detailed previously^40^ and is briefly summarized here. Mice were decapitated after deep anesthetization with isoflurane. The brain was removed and prepared for slicing in ice-cold modified ACSF (mACSF). The composition of the mACSF was (in mM): NaCl 124, NaHCO_3_ 26, KCl 3.2, MgSO_4_ 1, NaH_2_PO_4_ 0.5, MgCl_2_ 9, Glucose 10. The mACSF was bubbled with a gas mixture of 95% O_2_ and 5% CO_2_. Then, 400 µm-thick slices were cut with a vibratome in the presence of cold, oxygenated mACSF. The slices were allowed to recover for at least one hour at room temperature in a holding chamber filled with oxygenated, in vivo-like ACSF, whose composition was (in mM): NaCl 124, NaHCO_3_ 26, KCl 3.2, MgSO_4_ 1, NaH_2_PO_4_ 0.5, CaCl_2_ 1.1, and glucose 10. This ACSF was continuously bubbled with a 95% O_2_/5% CO_2_ gas mixture (pH 7.4).

### Ex vivo electrophysiological recordings of the brain slices

For the ex vivo recordings, a slice was transferred to a submersion-type chamber that was continuously gravity-fed with oxygenated (95% O_2_/5% CO_2_) in vivo-like ACSF at a flow rate of 3–3.5 ml/min. All recordings were performed at 34–35 °C. For recording, two probes were used, and 2 electrodes were tested for each probe. After deep insertion, one of the two probes was slowly pulled up in steps of 50–100 µm, yielding five pairs of recordings. Electrophysiological signals were amplified (×1000) and filtered (0.1 Hz–10 kHz) with a NeuroLog system (Digitimer, UK). Notably, 50 Hz noise was eliminated with a Humbug system (Quest Scientific, Canada). Low-pass filtering (0–100 Hz) and bandpass filtering (300–3000 Hz) were used offline to isolate LFP and spiking activity, respectively. All signals were digitized with a digitization rate of 20 kHz (power1401, CED, UK). The real-time display of the signals was achieved with an oscilloscope and Spike2 software (CED, UK). Spike sorting was conducted using Spike2. First, we computed the root mean square (RMS) of the voltage trace (running average of over 5 s). Next, 2 ms wide segments of voltage traces (“wavemarks”) were generated whenever negative deflections, likely corresponding to action potentials, exceeded a threshold set at −4 times the RMS. The wavemarks extended from −0.5 to +1.5 ms relative to the peak of the negative deflection. The minimal wavemark overlap was set at 0.5 ms. The wavemarks were then submitted to principal component analysis (PCA) and cluster analysis. Single units corresponded to clusters containing spikes of constant shape and displaying a refractory period of >1 ms were determined from interspike interval distribution histograms computed with a bin width of 0.2 ms.

### Packaging

Prior to recording, the flexible device was bonded to a customized flexible ribbon cable with golden traces (AXO-00021, pro-POWER, China) by using epoxy silver and photosensitive glue. The overall connection system was then passivated with insulating biocompatible glue (Polytec EP 601).

### In vivo implantation into a mouse brain

Male C57BL/6J mice aged 15–17 weeks, purchased from a commercial supplier (Charles River, Eculy, France), were used for the implantation tests in vivo. For anesthesia, subjects received a chemical induction of ketamine/xylazine (125/5 mg/kg) by intraperitoneal injection followed by gas induction with 3% vaporized isoflurane (TEM SEGA’s MiniHUB V3. Pessac). Once the mouse was placed on the surgical platform, anesthesia was maintained with isoflurane (1.5% v/v in O_2_). The level of anesthesia was checked by the lack of the withdrawal reflex after pinching the hindlimbs. A heating pad was positioned underneath the animal to keep the body temperature at 37 °C. Eye dehydration was prevented by topical application of ophthalmic gel. An incision was made through all skin layers at the top of the skull, the skin flaps were retracted, and the tissues detached from the bone. The skull was cleaned and dried with sterile cotton swabs, and a hole was drilled using a Harvard Apparatus MicroDrill (0.5 mm diameter: Les Ulis, France), allowing the dura mater to be carefully removed to expose the brain. The silk-parylene probes were inserted in the somatosensory (SS) cortex or visual (V1) cortex (SS stereotaxic coordinates: AP: −0.9, ML: −3.0; right V1 stereotactic coordinates: AP, −3.5 mm*;* ML*,* 2.4 mm; at a depth of 2 mm from the brain surface). All procedures above complied with the guidelines of State Scientific and Technological Commission Animal procedures and were approved by the national Animal Care and Ethics Committee (CE2A122 protocol number 2020051519454596) following European Directive 2010/63/EU.

## Supplementary information

Supporting material

Supplementary Material
