# Supplementary material for: Scalable batch fabrication of ultrathin flexible neural probes using a bioresorbable silk layer
Source: Microsyst Nanoeng. 2022 Feb 16;8:21. doi: 10.1038/s41378-022-00353-7 (PMC8847482; doi:10.1038/s41378-022-00353-7)
Supplement: Supplementary file 2 — Supplementary Material [file 41378_2022_353_MOESM2_ESM.docx]

Supplementary Material for:

Scalable Batch Fabrication of Ultrathin Flexible Neural Probes using a Bioresorbable Silk Layer

Clement Cointe ^1^, Adrian Laborde ^1^, Lionel G Nowak ^2^, Dina N Arvanitis,^3^ David Bourrier ^1^, Christian Bergaud^1^ and Ali Maziz*^1^

^1^ LAAS-CNRS, 7 Avenue du Colonel Roche, F-31400, Toulouse, France

^2^ CerCo, Université Toulouse 3, CNRS, Pavillon Baudot, CHU Purpan, BP 25202, 31052 Toulouse, France

^3^ UMR Institut National de la Santé et de la Recherche Médicale 1048, Institut des Maladies Métaboliques et Cardiovasculaires, Toulouse, France

**Probe microfabrication process:**

Step 1 (Fig S1.1) - Deposition of a cellulose acetate layer (≈2 μm) by spin-coating at 1000 rpm for 30 s (5 %wt.vol in acetone solution)

Step 2 (Fig S1.2) – Deposition of silk fibroin solution (7% wt.) by drop casting and drying at ambient conditions overnight, resulting in a 30-µm thick silk film

Step 3 (Fig S1.3) – Deposition of a 3 μm-thick base layer of Parylene C (PXC) onto the silk-coated substrate through CVD, using a C30S Comelec equipment

Step 4 (Fig S1.4 & Fig S2.A) - Shadow mask placement on the surface of the Parylene, held in place with a magnet

Step 5 (Fig S1.5) – Deposition by evaporation of a 50/200 nm layer of Ti/Au

Step 6 (Fig S1.6) - Metal patterning through the shadow mask

Step 7 (Fig S1.7) – Deposition of an another 1.3 μm top layer of Parylene C onto the processed metal layer

Step 8 (Fig S1.8) – Definition of the shape of the electrode pads and contacts by photolithography steps using a 2.6 µm ECI resist

Step 9 (Fig S1.9) - Reactive ion etching in O_2_ plasma 500 W and 20 mT

Step 10 (Fig S1.10) – Definition of the shape of the device body by photolithography step using a 20 µm NLOF resist followed by reactive ion etching in O_2_/CF_4_ plasma (75/25) 500 W and 20 mT

Step 11 (Fig S1.11 & Fig S2.B) - Dissolution of the cellulose acetate sacrificial layer in acetone and release of the bilayered silk-parylene probes

**Figure S1.** Schematic illustration of bilayered silk-parylene neural probes fabrication

**Figure S2. Silk-parylene neural probes fabrication overview. A)** Picture of the shadow mask used for metallization. **B)** Picture of two bilayered silk-parylene neural probes**.**

**Electrochemical cleaning and deposition**

To demonstrate the proper functioning of the electrodes and to characterize them, an electrochemical study was conducted. Before utilization, the electrodes must be electrochemically cleaned to ensure good deposition conditions. First, a series of 20 potential pulses (2 V vs. Ag/AgCl for 1s, -1 V vs. Ag/AgCl for 1s) was applied in PBS at room temperature. To probe the microelectrodes, a CV was performed in PBS at room temperature by potential sweeping between -0.3V and +1.3 V vs. Ag/AgCl reference at 200 mV/s.The activation of the microelectrodes made it possible to find a good electrochemical signature of gold (**Fig S3.A**). The improvement of the electrical properties was achieved by PEDOT:PSS deposition. CV was performed in EDOT:PSS solution (10 mM:34 mM) at room temperature by potential sweeping between -0.7 V and +1 V vs. Ag/AgCl reference at 10 mV/s (**Fig S3.B**).

**Figure S3. Electrochemical cleaning and characterizations of neural microelectrodes. A)** CV in PBS at 200 mV/s between -0.3 V and +1.3 V vs. Ag/AgCl reference after activation. B) PEDOT:PSS deposition by CV in EDOT:PSS solution at 10 mV/s between -0.7 V and +1 V vs. Ag/AgCl reference.

**Insertion test in mouse brain**


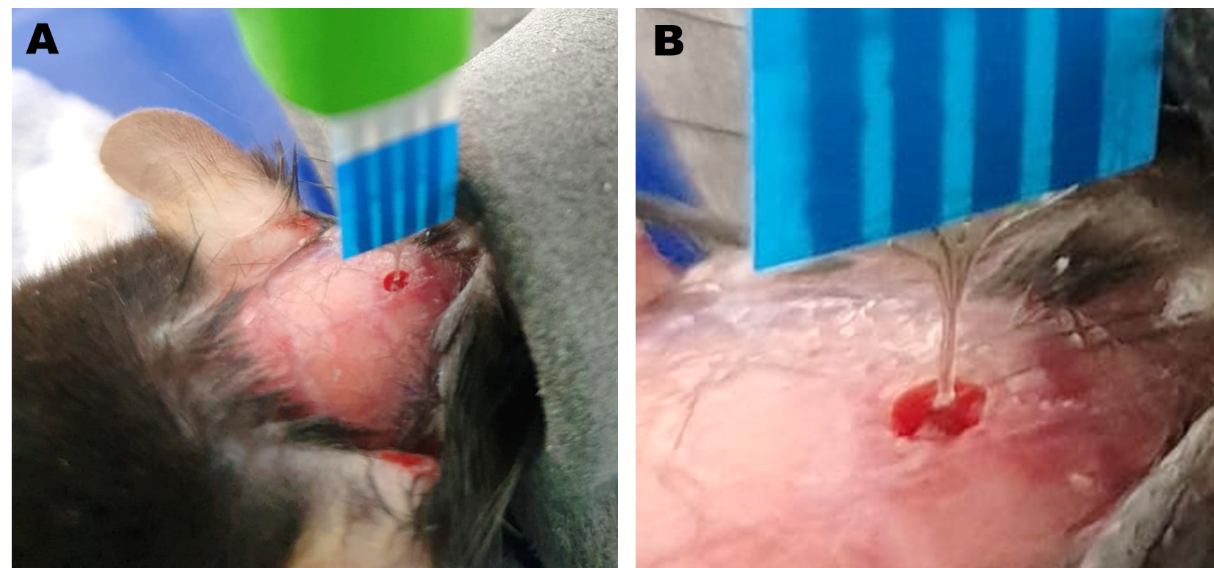


**Figure S4.** Picture of the probe after in-vivo implantation in mouse brain (pia matter)
